# Supplementary material for: Comparative Transcriptomic Profiling and Gene Expression for Myxomatous Mitral Valve Disease in the Dog and Human
Source: Vet Sci. 2017 Jul 17;4(3):34. doi: 10.3390/vetsci4030034 (PMC5644653; doi:10.3390/vetsci4030034)
Supplement: Supplementary file 1 [file vetsci-04-00034-s001.pdf]

veterinary sciences

MDPI

Review

Comparative Transcriptomic Profiling and Gene Expression for Myxomatous Mitral Valve Disease in the Dog and Human

Greg R. Markby [1,2], Kim M. Summers [1,2], Vicky E. MacRae [1,2] and Brendan M. Corcoran [1,2,*]

[1] Roslin Institute, The University of Edinburgh, Easterbush Veterinary Centre, Roslin, Scotland EH25 9RG, UK; s1029690@sms.ed.ac.uk (G.R.M.); kim.summers@roslin.ed.ac.uk (K.M.S.); vicky.macrae@roslin.ed.ac.uk (V.E.M.)

[2] Royal (Dick) School of Veterinary Studies, The University of Edinburgh, Easterbush Veterinary Centre, Roslin, Scotland EH25 9RG, UK

* Correspondence: Brendan.Corcoran@ed.ac.uk; Tel.: +44-131-6507-650; Fax: +44-131-6507-652

Academic Editors: Sonja Fonfara and Lynne O'Sullivan

Received: 1 March 2017; Accepted: 6 June 2017; Published: 17 July 2017

Abstract: Myxomatous mitral valve disease is the single most important mitral valve disease in both dogs and humans. In the case of the dog it is ubiquitous, such that all aged dogs will have some evidence of the disease, and for humans it is known as Barlow's disease and affects up to 3% of the population, with an expected increase in prevalence as the population ages. Disease in the two species show many similarities and while both have the classic myxomatous degeneration only in humans is there extensive fibrosis. This dual pathology of the human disease markedly affects the valve transcriptome and the difference between the dog and human is dominated by changes in genes associated with fibrosis. This review will briefly examine the comparative valve pathology and then, in more detail, the transcriptomic profiling and gene expression reported so far for both species.

Keywords: canine; human; myxomatous mitral valve disease; Barlow's disease; transcriptomic profiling

1. Introduction

Myxomatous mitral valve disease (MMVD) is the most important acquired cardiac disease of the dog, with almost all dogs developing some form of the disease if they live long enough [1]. MMVD is also described in humans with 2%–3% of the global population estimated to have the disease and approximately 15% of those affected requiring valve surgery [2–4]. The disease has previously been reported to share many similarities between the two species, with the dog suggested as a potential model for the human disease [1,5,6]. However there are important differences in pathological appearance and these differences are also evident in the gene changes found with transcriptomic profiling [2,7–10]. This review will examine the similarities and differences between MMVD in the dog and human, with a particular emphasis on the gene changes identified on transcriptomic profiling and real time polymerase chain reaction (RT-PCR). To date there is one study using RNA sequencing in the dog and none for human MMVD [11].

2. Comparative Pathology

When comparing canine and human MMVD it is the difference in pathology that is the most striking. The normal mitral valve in both species is reported to be relatively similar in structure [5]. Briefly, the valve consists of an outer layer of valvular endothelial cells (VECs) which are anchored to the internal valve structure by a basement membrane consisting of proteins such as the laminins

(LAMA1-5), the nidogens (NID1 & 2), type IV collagens and basement membrane-specific heparan sulfate proteoglycan [12]. Towards the interior of the valve on the atrial side there is a highly organised, but thin, layer of elastin fibres and mature collagen forming the atrialis, and this layer has been shown to support valve movement through extension and recoil [13]. Beneath the atrialis is the spongiosa which contributes to the valve structure and compressibility and primarily consists of a mixture of proteoglycans (large and small) and glycosaminoglycans (GAGs) (in particular hyaluronan) which covalently attach to proteoglycans, some free collagen fibres and valvular interstitial cells (VICs), which express extracellular matrix components (ECM) [13,14]. Valvular interstitial cells (VICs) in normal valves are for the most part quiescent so only operate in a homeostatic role [14–16]. Towards the ventricular side of the valve is the fibrosa layer which is made up of densely packed collagen fibres (types I, III and V mainly) with an intermingling of some elastin closer to the outer endothelial layer (separately sometimes termed the ventricularis). The fibrosa and ventricularis together have a more tensile role, protecting against prolapse from the strong hemodynamic forces during ventricular ejection [13].

When the disease appears (which has a close association with age) and then progresses, there are some similarities between dogs and humans. Myxomatous degeneration is typified by a disorganization and dysregulation of the extracellular matrix of the valve, with expansion of the spongiosa and loss of the fibrosa, dysfunction and potential loss of some of the VECs, and activation of the VICs from a quiescent to an activated-myofibroblast phenotype [14,15,17–22]. It is activation of the VICs which is thought to play a pivotal role in the development of disease through the release of catabolic enzymes and the dysregulation of proteoglycan and collagen synthesis resulting in aberrant remodelling [15,18,23]. In areas of myxomatous degeneration expansion of the spongiosa (containing activated VICs) is observed with increased presence of GAGs, proteoglycans, disorganized collagen fibrils as well as increased retention of water [14,21,23–26]. In both the atrialis and fibrosa there is also a breakdown of the ordered collagen and elastin layers, and all this contributes to the eventual problem of valvular incompetency [23]. Lastly, while MMVD can affect both leaflets in the dog, it mainly affects the posterior leaflet in humans [6,27,28]. These changes are summarised in Figure 1.

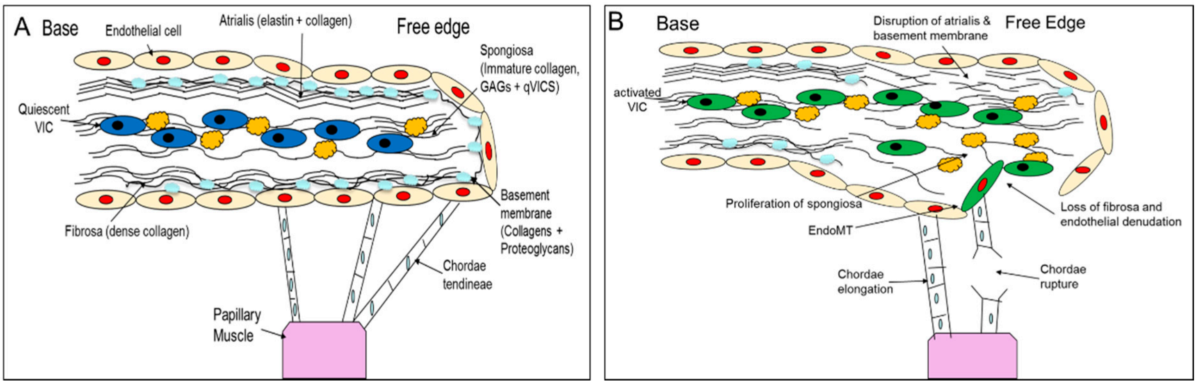

Figure 1. Schematic diagram summarising pathological changes seen in myxomatous degeneration. (**A**) Illustrates the normal valve structure and cellular composition. (**B**) Pathological changes associated with myxomatous disease; not shown is the development of fibrosis in the atrialis and fibrosa of the human valve.

A clear contrast between human (Barlow's Disease) and canine MMVD is the appearance of extensive fibrosis in the former [22,29,30]. Fibrosis of human valves occurs on both the atrial and ventricular surface and encases a central myxomatous core. The fibrosis forms so called "over-lays" on the atrial surface which tends to be more severely affected [22,30]. In addition, complicating the human picture is that several diseases including rheumatic fever, bacterial endocarditis and fibroelastic deficiency (FED) cause valve changes, and the term mitral valve prolapse (MVP) is often used without referencing the disease which caused the MVP. For these reasons clinical phenotype has to be carefully considered when evaluating for gene expression in human mitral valve disease.

FED contrasts with Barlow's disease in that it has relatively fast onset (months) and is the result of deficiency in key connective tissue components including collagens, proteoglycans and elastin with chordae rupture being a common occurrence, for this reason Barlow's disease is more analogous with canine MMVD [2,31,32]. Greenhouse and others [2] report the transcriptome for human MVP, but define that as FED. Overall, the myxomatous changes appear to be similar in the dog and human, but while studies have effectively evaluated the transcriptome of myxoid degeneration in the dog, this has not been possible in human MMVD due to the preponderance of fibrosis [5,8,10,21,23,33]. There are limited data on the cellular changes that might be involved in or contributing to the fibrotic response in human valves.

3. Gene Changes in Canine and Human MMVD

There are limited amounts of data on the valve transcriptome and specific gene changes, as assessed by RT-PCR, in both dogs and humans, but enough data to show consistent changes that allow hypothesis driven studies examining putative signalling pathways contributing to disease pathogenesis, and to identify those genes active in end-stage disease. There are marked differences in gene expression between the species, but there are also certain caveats that need to be considered for the studies reported to date. For the dog, identifying age-matched normal controls is nearly impossible since all dogs have some evidence of the disease as they age. For human MMVD problems arise with accurate phenotyping due to the heterogeneity of degenerative valvulopathies, and what tissue is sampled is based on the patient's clinical need. Not surprisingly, reports have been primarily on advanced stage disease with limited data on changes as disease progresses in both species. An additional confounding factor is that most patients (in both species) will likely have been on cardiac medications, the effects of which on valve gene expression are currently unknown.

Transcriptomic profiling with bioinformatic analysis allows identification of gene signal intensity, gene changes between groups, gene ontology enrichment, network identification, functional clustering and biological effect annotation. This can give powerful insight into gene function in disease, but needs to be interpreted with some caution. Proof that genes are actually expressed requires RT-PCR validation and that in itself does not confirm that there is protein translation. Nevertheless, transcriptomic profiling indicates relevant signalling pathways that can be then interrogated in greater detail and functionally assessed.

Varying numbers of genes have been identified to be differentially expressed in both the dog and human and the numbers reported depend on finalized intensity cut-off using fold change (typically > 1.5) and false discovery rate (FDR < 0.05) [2,7–10,34]. For the reports by Thalji and others (human) and Lu and others (canine) 2602 and 5397 differentially expressed genes were found respectively using the same FDR and fold change [8,10]. With further filtering based on signal intensity, but not reported for the human transcriptome, Lu and others reduced the number to 591 differentially expressed annotated canine genes [8]. Comparison between these two studies is shown in Tables S1 and S2. In the single study that used high-throughput RNA sequencing in the dog, 263 genes were found to be differentially expressed [11].

While differential expression is clearly important in any gene study, the intrinsic gene expression can also be of interest (as assessed by signal intensity), but is rarely reported, and within certain families of genes there can be high signal intensity genes indicting their importance in tissue homeostatic control. Examples for the dog include genes important in tissue structure such as collagen genes *COL 1A1, 2* & *3, 12A1* and *14A1* as well as the proteoglycans lumican (LUM) and versican (VCAN). Further exemplifying this point is the high expression of the matrix-metalloproteinases (MMP) *MMP2, 14* and *19* and their tissue inhibitors (TIMP) *TIMP2* and *3* which are required for control of the ECM, and there are many more examples [8].

For MMVD, dogs and humans do share gene changes based on gene ontology and KEGG (Kyoto Encyclopedia of Genes and Genomes) pathway analysis. For example, ECM, cell signalling, cell movement, cardiovascular development, inflammation and endothelial-to-mesenchymal transition (EndoMT), but with some variation between studies [2,7–11,34]. Many of these functional changes are not surprising considering the pathology of MMVD, not least affecting the ECM, but others such

as inflammation likely represent signalling pathways involved in a whole range of biological functions.

In the canine MMVD transcriptome there is consistent increased expression of inflammatory genes, but this is not consistently reported in human MMVD [2,7–9,11,34]. There is a small increase in macrophage and mast cell number in canine MMVD, which is not seen in human valves, but is located towards the valve base and not in areas affected by disease [6,16,35,36]. In the inflammation category the main gene changes are in the expression of toll-like receptors and interleukins, which are involved in both the control of inflammation as well as other biological pathways. Changes in these two gene families are observed in both human and canine profile sets but do not, in many cases, show matching changes in specific genes [8,10]. This may be indicative of differences in the phenotypic changes in the VIC and VEC populations in both disease [37]. However, a better understanding of the phenotypic switch of VICs, as well as the potential role of EndoMT in VECs, is required before any conclusions can be drawn.

Endothelial-to-mesenchymal transition (EndoMT) occurs in canine MMVD, and in induced ovine models of the disease, with increased co-expression of the myofibroblast marker alpha-smooth muscle actin; α-SMA (encoded by the *ACTA2* gene) and the endothelial cell marker platelet endothelial cell adhesion molecule 1; CD31 (*PECAM1*) in endothelial cells that have migrated into the valve stroma [38,39]. It is hypothesized that this also occurs in human MMVD, but has yet to be confirmed. Comparing the transcriptomic data for both species suggests EndoMT does occur in humans MMVD. There is decreased gene expression for the key basement membrane components *NID1* and *LAMA2* in both species and decreased gene expression of *COLI4A1, A2* and *A6* in humans, all of which will allow for greater migratory potential of endothelial cells [8,10]. This is reflected in a loss of endothelial cells in humans similar to that seen in dogs during the disease [17,36]. Additionally, downregulation of the cell-cell adhesion glycoprotein vascular endothelial cadherin gene (*CDH5*) and increased expression of the pro-migratory protein hyaluronic synthase 2 (*HAS2*) [8,10,34], required for EndoMT, has been shown in both species, and confirmed by immunohistochemistry (IHC) in the dog [38]. The mechanisms underpinning activation of EndoMT have yet to be identified, but NOTCH signalling appears not to be involved in the dog [38].

There are additional endothelial-related gene changes that are different between the two species. Canonical pathways involved in endothelial function are activated in the dog, including endothelin-1 signalling, but several endothelium-related genes are typically downregulation in human MMVD [10,38]. Increased nitric oxide synthase expression is found in canine MMVD and is important in regulating oxidative stress, and reactive oxygen species are changed in human MMVD [11,40]. However, while the role of endothelial damage and dysfunction is well recognised in the dog, this has not yet been comprehensively reported in human MMVD [19,20,41,42].

Not surprisingly ECM gene changes are important features of the disease transcriptome in both species. Despite the well characterised pathological changes observed in MMVD, the precise underlying molecular mechanisms responsible for disease development remain unknown [15,16,18,29,43]. ECM remodelling is presumed to be due to phenotypic transition of quiescent VICs into an activated myofibroblast phenotype. This change in phenotype can, as previously mentioned, be measured by increased expression of α-SMA, identified by IHC and RT-PCR [15,16,18,44]. Paradoxically, to date increased α-SMA mRNA expression has not been shown by transcriptomic profiling in either canine or human MMVD [9–11,34,38,45]. Regardless of this, activation of VICs to a myofibroblastic phenotype is thought to be critical to disease development and understanding the cause of this activation is of major research interest.

Several gene families and signalling pathways have been implicated not only in this VIC activation process, but also in other aspects of disease including EndoMT, metalloprotease production and dysregulation of ECM protein synthesis [2,7,8,10,40,45–47]. Of particular interest are the transforming growth factor-β (TGF-β), the renin-angiotensin and serotonergic signalling pathways. Disease links to these have been identified both through focused histopathological and gene studies as well as transcriptome wide analysis. As previously mentioned, comparison between dog and human is complicated by the fibrotic component of human MMVD, such that the human

transcriptome is dominated by the TGF-β signalling pathway, presumably driving fibrosis through canonical Suppressor of mothers against decapentaplegic (SMAD) signalling [40].

The TGF-β superfamily itself contains many structurally similar proteins including the TGF-β subfamily (TGF-β1-3) and the bone morphogenetic proteins (BMPs) [7,48]. BMPs, particularly BMP2 and 4, play a role in development of the valve cusp [49,50]. Their importance in valve development implicates them in disease as they regulate many of the changes seen such as ECM modification and EndoMT activation. BMP4 is upregulated, alongside the downstream signalling components Cartilage oligomeric matrix protein (COMP) and *SOX9*, in human MMVD valves [34]. In the dog BMP6 is consistently upregulated [8,9]. The BMPs function through SMAD and non-SMAD (extracellular regulated protein kinases (ERK) 1/2 etc.) pathways to elicit transcriptional and non-transcription effects that modify cell actin organization, and cell migration, survival and differentiation, which are all cardinal features of MMVD [15,16,18,20,40,49–51].

With respect to TGF-β1 and 2 signalling there are distinct differences currently reported comparing canine and human MMVD. The TGF-β signalling pathway has a major role in myofibroblast differentiation, EndoMT and alteration in ECM protein and MMP activity in myocardial disease and cardiac fibrosis [52,53]. In dogs, currently no significant changes in gene expression assessed by transcriptomic profiling of the TGF-β superfamily have been identified, except in the expression of ENG (endoglin, part of the TGF-β receptor complex) and BMP6 mentioned above [8,9,11]. This contrasts with protein and gene expression measured by IHC and RT-PCR showing variably increased expression of TGF-β1, 2 and 3 and TGF-β2 receptor in diseased canine valves [46,54,55]. Furthermore, assessment of canine VICs in culture has shown that exogenous TGF-β1 can cause myofibroblast differentiation, as in human VICs, suggesting a role in disease [56,57]. Contrasting to the dog, transcriptomic findings for TGF-β family members in human valves seem to be more consistent. There is a consistent upregulation in *TGF-β2* and it is widely thought that this upregulation causes the remodelling seen in human MMVD [2,7,10,34,45]. Local increase in the expression of *TGF-β1*, using RT-qPCR, has also been reported [40]. These findings in human valves are expected, considering the fibrotic component of the disease. The downstream signalling changes, including increased expression of *MMP2*, connective tissue growth factor (*CTGF*), and the SMAD-specific ligases SMURF1 and 2, as well as increased phosphorylated SMAD2/3, further support the idea that TGF-β1 canonical pathway signalling drives fibrosis in human MMVD, with an associated increase in collagen types I and III [40,58]. At present it is problematic to explain the role of TGF-β in canine MMVD, except for the decreased expression of *ENG* which can dampen the pro-fibrotic effects of TGF-β, and further work is need in this area [8].

There is, however, consistently reported increased expression of the 5-HT (5-hydroxytryptamine or serotonin) receptor genes *5HTR2B* in dog and *5HTR2A* in human MMVD, as well as an increase in the serotonergic pathway rate limiting enzyme tryptophan hydroxylase 1 (TPH1) in human and dog mitral valves, and an increase in *TPH1* gene expression in canine MMVD [8–10,59]. Serotonergic signaling has been associated with a range of valvulopathies and can affect TGF-β signalling. 5HT can induce VIC proliferation and ECM production through the activation of ERK1/2 pathways, but has not been shown to increase α-SMA expression in the same cells [60,61]. Likewise angiotensin 2 (AGT2) has been associated with disease, via renin-angiotensin signalling, through its known role in cardiac remodelling hypertrophy and induction of some aspects of MMVD in a mouse model [10,62]. Inhibiting AGT2 signalling not only improves MMVD outcomes in dogs, but also attenuates TGF-β signalling in human VICs [63,64]. However, no direct changes in protein or gene expression of members of the angiotensin family have been found in MMVD in either species. Interestingly, interactions between the AGT2 type 1 receptor (AT1) and 5HTR2B receptor have previously been suggested, and combined stimulation with 5HT and AGT2 in both a mouse model of MMVD and porcine cultured VICs induces valve remodelling and increases expression of α-SMA [65,66]. It may be that MMVD requires similar co-regulation by different factors in order to progress MMVD and initiate ECM dysregulation.

Important in the control of ECM production and homeostasis are the MMP and ADAMTS (A Disintegrin and Metalloprotease with Thrombospondin Motifs) metalloproteases and their inhibitors

TIMPs. MMP and TIMP protein and gene expression in diseased valves have been reported, but the findings are inconsistent [6,8,9,11,15,67]. The inconsistency in valve expression may reflect remodelling activity at the time of sampling as MMPs (and therefore TIMP expression) can be transient, but MMPs likely have some role in valve remodelling in disease [68]. Further contrasts between species have been reported with the elastin degrading proteases MMP2 and 9, showing increased protein expression and activity in humans [69], but no change in MMP9 and reduced MMP2 in diseased canine valves [70]. Interestingly an increase in mRNA for a different elastin degrading protease *MMP12* has been found in the dog transcriptome [8], but not in human. There are significant decreases in *MMP14* and *16* expression in the dog, both important in degradation of several ECM components, but increased expression of *MMP16* in human valves [8,10,68]. The ADAMTS family of genes, like the MMPs, have a wide variety of effects on the ECM through the cleavage of substrates and have been shown to be affected in disease of both species. In human MMVD there is downregulation of *ADAMTS1*, *5* and *9* [10]. These proteases are involved in the cleavage of the proteoglycans aggrecan and versican, as well as some other ECM components, and knockout and haplo-insufficient mouse models of *ADAMTS5* and *9*, respectively, have shown a MMVD phenotype [10,71–73]. Increased proteoglycan deposition with MMVD is seen in both species. There is increased gene expression for versican in human MMVD and downregulation of *ADAMTS1*, *5* and *9* which would contribute to increased versican deposition [1,10,26,34,74]. In human valves there is upregulation of *ADAMTS6* which currently has no known substrate [10]. In the dog, there is significant downregulation of *ADAMTS2*, *19* and *L4*, with ADAMTS2 and L4 having roles in procollagen maturation and fibrillin-1 binding respectively, whereas the function of ADAMTS19 is currently unknown [8,71,75]. Reduced collagen turnover is recognised in diseased canine valves and the downregulation in *ADAMTS2* may be responsible for this [19,24,76]. For the small leucine-rich proteoglycans (SLRP) many members show high intensity gene expression in canine mitral valves, but only chondroadherin (*CHAD*) and keratocan (*KERA*) have so far been shown to be significantly downregulated, while *HAPLN1* (hyaluronan and proteoglycan link protein 1) is upregulated in human and downregulated in canine diseased valves [8,10,34,77]. In human MMVD, in contrast to the dog, there is a tendency for increased SLRP protein expression, including decorin, biglycan (and the proteoglycan versican mentioned above) [26,78]. Proteoglycans are fundamentally important in the synthesis and organization of the spongiosa and fibrosa, derangement of both which are cardinal features of MMVD.

There are many additional gene changes in MMVD that encompass a wide variety of biological functions that would be too extensive to review here in detail. Examples include, genes associated with oxidative stress, ossification, epithelial (endothelial) proliferation and potential up-stream regulators such as interleukins, interferons, tumor necrosis factor (TNF), nuclear factor kappa-light-chain-enhancer of activated B cells (NFκB), low density lipoprotein receptor (LDLR), and the heat shock proteins (HSP). There is some evidence for the role of reactive oxygen species (ROS) in human MMVD, with increased expression of nicotinamide adenine dinucleotide phosphate-oxidase (NOX) 2 and 4, and this has also been identified in cultured valve cells from superoxide dismutase (SOD1) knockout mice where there is upregulation of pro-fibrotic ROS associated genes [40]. Marked downregulation of several members of the metallothionein family of genes in human valves, which contrasts with reports of upregulation in the dog [9,11], is further evidence of oxidative stress in human MMVD [10].

Lastly, there is limited data on the differential expression of non-coding RNAs (ncRNAs) in valve tissue. NcRNAs are fundamentally important in controlling gene expression. MicroRNAs, a class of ncRNAs, act to maintain intracellular homeostasis and essentially "buffer" against rapid changes in gene expression and cell metabolism. Their dysregulation is believed to have a fundamental role in disease pathogenesis. Differential expression of microRNAs (miRs) has been reported in humans comparing MMVD and FED [79], and changes in expression of 29 canine (pre-) miRs were mentioned in one transcriptomic study [8]. In the dog there is evidence for a range of differentially expressed miRs, all downregulated, but some with very low signal intensity requiring cautious interpretation (C.C. Lu, pers.comm). Some miRs have high signal intensity indicating robust

epigenetic control of genomic expression in normal valves. One of the most striking changes is downregulation of *miR-29*, which has extensive regulatory functions controlling ECM genes. Additional altered miRs in the canine valve include *218*, *Let7c*, *214*, *30* and *15*, which have varied effects on regulation of genes associated with ECM, cell migration, differentiation and EndoMT. In human MMVD miR changes have been reported when compared to FED, which is dominated by elastin fibre infiltration, but not to normal valves. There were 20 miRs significantly differentially expressed between the two diseases. Target prediction software identified various ECM genes regulated by the differentially expressed miRs and differences in those ECM genes were found for several SLRPs [79]. Much further work is needed on the role of miRs and other non-coding RNAs in canine and human MMVD.

4. Conclusions

To date there are a limited number of transcriptomic profiling studies for canine and human MMVD, often with low sample numbers, making the drawing of categorical conclusions problematic. More data, as well as data from a wider range of stages of disease, are needed to increase interpretative confidence and to understand disease pathogenesis in full. Nevertheless, the current data gives an insight into the signalling pathways involved in the MMVD, and so allows for more focused hypothesis and mechanism-driven studies examining function. Furthermore, although there is a similar gross pathology, and some of those similarities are carried through to molecular differences, the transcriptomic data clearly show differences between canine and human MMVD that reflect the contribution of fibrosis to end-stage disease pathology in humans. Additional studies will be needed to examine local tissue gene expression and protein translation, to address why dogs do not develop fibrosis and humans do, and hence the validity of the dog disease as a model of human MMVD.

Supplementary Materials: The following are available online at www.mdpi.com/2306-7381/4/3/34/s1, Table S1, Partial comparison of gene fold changes in human MMVD (Thalji et al. 2015 [10]; 2602 differentially expressed genes) with the 591 genes reported in the dog by Lu et al. 2015 [8]. Both studies used the same FDR and fold settings, but Lu et al. also use signal intensity to derive a much smaller gene set from an original set of 5397 (not compared) differentially expressed genes. Only 83 differentially expressed genes were reported in both data sets of which 40 showed the same direction of fold change (+ or −) and 43 had the opposite fold change. The data illustrates that while there are similarities, there are also clear difference between human and canine MMVD at the transcriptomic level. The full gene names are shown, Table S2. Some illustrative examples of fold change for a selection of genes in human and canine MMVD, illustrating similar and dissimilar changes in both species. 0 = no report of gene change. The genes selected represent signalling pathways involved in cell differentiation, endothelial-to-mesenchymal transition, extra-cellular matrix and inflammation. Of particular note are the differences in genes associated with myofibroblast activation.

Acknowledgements: G.R. Markby The Dogs Trust; K.M. Summers BBSRC BB/J004316/1; V.E. MacRae; BBSRC BB/J004316/1

Conflicts of Interest: The authors declare no conflict of interest.

Abbreviations

MMVD:	Myxomatous mitral valve disease
RT-PCR:	Real time polymerase chain reaction
VEC:	Valvular endothelial cell
LAMA:	Laminin
NID:	Nidogen
GAG:	Glycosaminoglycan
VIC:	Valvular interstitial cell
ECM:	Extracellular matrix
FED:	Fibroelastic deficiency
MVP:	Mitral valve prolapse
FDR:	False discovery rate

COL:	Collagen
LUM:	Lumican
VCAN:	Versican
MMP:	Matrix-metalloproteinase
TIMP:	Tissue inhibitor of metalloproteinase
KEGG:	Kyoto Encyclopedia of Genes and Genomes
EndoMT:	Endothelial-to-mesenchymal transition
α-SMA:	Alpha-smooth muscle actin
CD31:	Platelet endothelial cell adhesion molecule 1
CDH5:	Vascular endothelial cadherin
HAS2:	Hyaluronic synthase 2
IHC:	Immunohistochemistry
TGF-β:	Transforming growth factor–β
SMAD:	Suppressor of mothers against decapentaplegic
BMP:	Bone morphogenetic proteins
COMP:	Cartilage oligomeric matrix protein
ENG:	Endoglin
TGFBI:	TGF-β-induced
CTGF:	Connective tissue growth factor
5-HT:	5-hydroxytryptamine
TPH1:	Tryptophan hydroxylase 1
AGT2:	Angiotensin 2
AT1:	AGT2 type 1 receptor
ADAMTS:	A Disintegrin And Metalloproteinase with Thrombospondin Motifs
SLRP:	Small leucine-rich proteoglycans
CHAD:	Chondroadherin
KERA:	Keratocan
HAPLN1:	Hyaluronan and proteoglycan link protein 1
TNF-α:	Tumor necrosis factor α
NFκB:	Nuclear factor kappa-light-chain-enhancer of activated B cells
LDLR:	Lipoprotein receptor
HSP:	Heat shock proteins
ROS:	Reactive oxygen species
NOX:	Nicotinamide adenine dinucleotide phosphate-oxidase
SOD:	Superoxide dismutase
ncRNAs:	Non-coding RNAs
miRs:	MicroRNAs

References

1. Connell, P.S.; Han, R.I.; Grande-Allen, K.J. Differentiating the aging of the mitral valve from human and canine myxomatous degeneration. *J. Vet. Cardiol.* **2012**, *14*, 31–45.
2. Greenhouse, D.G., A. Murphy, P. Mignatti, J. Zavadil, A.C. Galloway, and L.B. Balsam, Mitral valve prolapse is associated with altered extracellular matrix gene expression patterns. *Gene* **2016**, *586*, 56–61.
3. Freed, L.A., D. Levy, R.A. Levine, M.G. Larson, J.C. Evans, D.L. Fuller, B. Lehman, and E.J. Benjamin, Prevalence and clinical outcome of mitral-valve prolapse. *N. Engl. J. Med.* **1999**, *341*, 1–7.
4. d'Arcy, J.L., B.D. Prendergast, J.B. Chambers, S.G. Ray, and B. Bridgewater, Valvular heart disease: the next cardiac epidemic. *Heart* **2011**, *97*, 91–93.
5. Pedersen, H.D.; Haggstrom, j. Mitral valve prolapse in the dog: a model of mitral valve prolapse in man. *Cardiovasc. Res.* **2000**, *47*, 234–243.
6. Aupperle, H. and S. Disatian, Pathology, protein expression and signaling in myxomatous mitral valve degeneration: Comparison of dogs and humans. *J. Vet. Cardiol.* **2012**, *14*, 59–71.

7. Hulin, A., C. Deroanne, C. Lambert, J.O. Defraigne, B. Nusgens, M. Radermecker, and A. Colige, Emerging pathogenic mechanisms in human myxomatous mitral valve: lessons from past and novel data. *Cardiovasc. Pathology* **2013**, *22*, 245–250.
8. Lu, C.C.; M.M. Liu; G. Culshaw; M. Clinton; D.J. Argyle; B.M. Corcoran. Gene network and canonical pathway analysis in canine myxomatous mitral valve disease: A microarray study. *Vet. J.*, **2015**, *204*, 23–31.
9. Oyama, M.A.; Chittur, S.V. Genomic expression patterns of mitral valve tissues from dogs with degenerative mitral valve disease. *Am. J. Vet. Res.* **2006**, *67*, 1307–1318.
10. Thalji, N.M.; Hagler, M.A.; Zhang, H.; Casaclang-Verzosa, G.; Nair, A.A.; Suri, R.M.; Miller, J.D. Nonbiased Molecular Screening Identifies Novel Molecular Regulators of Fibrogenic and Proliferative Signaling in Myxomatous Mitral Valve Disease. *Circ. Cardiovasc Genet.*, **2015**, *8*, 516–528.
11. Li, Q.; Freeman, L.M.; Rush, J.E.; Huggins, G.S.; Kennedy, A.D.; Labuda, J.A.; Laflamme, D.P.; Hannah, S.S. Veterinary Medicine and Multi-Omics Research for Future Nutrition Targets: Metabolomics and Transcriptomics of the Common Degenerative Mitral Valve Disease in Dogs. *OMICS*, **2015**, *19*, 461–470.
12. Hallmann, R.; Horn, N.; Selg, M.; Wendler, O.; Pausch, F.; Sorokin, L.M. Expression and function of laminins in the embryonic and mature vasculature. *Physiol Rev.*, **2005**, *85*, 979–1000.
13. Hinton, R.B.; Yutzey, K.E. Heart Valve Structure and Function in Development and Disease. *Ann. Rev. Physiology 73*, **2011**, *73*, 29–46.
14. Levine, R.A.; Hagege, A.A.; Judge, D.P.; Padala, M.; Dal-Bianco, J.P.; Aikawa, E.; Beaudoin, J.; Bischoff, J.; Bouatia-Naji, N.; Bruneval, P.; *et al.*, Mitral valve disease—Morphology and mechanisms. *Nat. Rev. Cardiol*, **2015**, *12*, 689–710.
15. Disatian, S.; Ehrhart, E.J., 3rd; Zimmerman, S.; Orton, E.C. Interstitial cells from dogs with naturally occurring myxomatous mitral valve disease undergo phenotype transformation. *J. Heart Valve. Dis*. **2008**, *17*, 402–411.
16. Han, R.I.; Black, A.; Culshaw, G.J.; French, A.T.; Else, R.W.; Corcoran, B.M. Distribution of myofibroblasts, smooth muscle-like cells, macrophages, and mast cells in mitral valve leaflets of dogs with myxomatous mitral valve disease. *Am. J. Vet. Res.* **2008**, *69*, 763–769.
17. Corcoran, B.M.; Black, A.; Anderson, H.; McEwan, J.D.; French, A.; Smith, P.; Devine, C. Identification of surface morphologic changes in the mitral valve leaflets and chordae tendineae of dogs with myxomatous degeneration. *Am. J. Vet. Res.* **2004**, *65*, 198–206.
18. Rabkin, E.; Aikawa, M.; Stone, J.R.; Fukumoto, Y.; Libby, P.; Schoen, F.J. Activated interstitial myofibroblasts express catabolic enzymes and mediate matrix remodeling in myxomatous heart valves. *Circulation* **2001**, *104*, 2525–2532.
19. Black, A.; French, A.T.; Dukes-McEwan, J.; Corcoran, B.M. Ultrastructural morphologic evaluation of the phenotype of valvular interstitial cells in dogs with myxomatous degeneration of the mitral valve. *Am. J. Vet. Res.*, **2005**, *66*, 1408–1414.
20. Han, R.I.; Clark, C.H.; Black, A.; French, A.; Culshaw, G.J.; Kempson, S.A.; Corcoran, B.M. Morphological changes to endothelial and interstitial cells and to the extra-cellular matrix in canine myxomatous mitral valve disease (endocardiosis). *Vet. J.* **2013**, *197*, 388–394.
21. Fox, P.R. Pathology of myxomatous mitral valve disease in the dog. *J. Vet. Cardiol*. **2012**, *14*, 103–126.
22. Roberts, W.C.; Vowels, T.J.; Ko, J.M.; Hebeler, R.F. Gross and Histological Features of Excised Portions of Posterior Mitral Leaflet in Patients Having Operative Repair of Mitral Valve Prolapse and Comments on the Concept of Missing (= Ruptured) Chordae Tendineae. *J. Amer. College Cardiol.* **2014**, *63*, 1667–1674.
23. Han, R.I.; Black, A.; Culshaw, G.; French, A.T.; Corcoran, B.M. Structural and cellular changes in canine myxomatous mitral valve disease: An image analysis study. *J. Heart Valve Dis*, **2010**, *19*, 60–70.
24. Hadian, M.; Corcoran, B.M.; Bradshaw, J.P. Molecular changes in fibrillar collagen in myxomatous mitral valve disease. *Cardiovasc Pathol* **2010**, *19*, e141–148.
25. Grande-Allen, K.J.; Griffin, B.P.; Ratliff, N.B.; Cosgrove, D.M.; Vesely, I. Glycosaminoglycan myxomatous mitral profiles of leaflets and chordae parallel the severity of mechanical alterations. *J. Amer. College Cardiol.* **2003**, *42*, 271–277.
26. Gupta, V.; Barzilla, J.E.; Mendez, J.S.; et al. Abundance and location of proteoglycans and hyaluronan within normal and myxomatous mitral valves. *Cardiovasc. Pathol.* **2009**, *18*, 191–197.
27. Lester, W.M. Myxomatous mitral valve disease and related entities: The role of matrix in valvular heart disease. *Cardiovasc Pathol.* **1995**, *4*, 257–264.

28. Davies, M.J.; Moore, B.P.; Braimbridge, M.V. The floppy mitral valve. Study of incidence, pathology, and complications in surgical, necropsy, and forensic material. *Br. Heart J.* **1978**, *40*, 468–481.
29. Buchanan, J.W. Chronic valvular disease (endocardiosis) in dogs. *Adv. Vet. Sci Comp. Med.* **1977**, *21*, 75–106.
30. McDonald, P.C.; Wilson, J.E.; McNeill, S.; Gao, M.; Spinelli, J.J.; Rosenberg, F.; Wiebe, H.; McManus, B.M. The challenge of defining normality for human mitral and aortic valves: geometrical and compositional analysis. *Cardiovasc. Pathol*. **2002**, *11*, 193–209.
31. Matsumaru, I.; Eishi, K.; Hashizume, K.; Kawano, H.; Tsuneto, A.; Hayashi, T. Clinical and pathological features of degenerative mitral valve disease: billowing mitral leaflet versus fibroelastic deficiency. *Ann. Thorac Cardiovasc Surg*. **2014**, *20*, 987–994.
32. Anyanwu, A.C.; Adams, D.H. Etiologic classification of degenerative mitral valve disease: Barlow's disease and fibroelastic deficiency. *Semin Thorac Cardiovasc Surg*. **2007**, *19*, 90–96.
33. Han, R.I.; Impoco, G.; Culshaw, G.; French, A.T.; Black, A.; Corcoran, B.M. Cell maceration scanning electron microscopy and computer-derived porosity measurements in assessment of connective tissue microstructure changes in the canine myxomatous mitral valve. *Vet. J.* **2013**, *197*, 502–505.
34. Sainger, R., Grau, J.B.; Branchetti, E.; Poggio, P.; Seefried, W.F.; Field, B.C.; Acker, M.A.; Gorman, R.C.; J H Gorman, 3rd; Hargrove, C.W., 3rd, *et al.* Human myxomatous mitral valve prolapse: role of bone morphogenetic protein 4 in valvular interstitial cell activation. *J. Cell. Physiol*, **2012**, *227*, 2595–2604.
35. Stein, P.D.; Wang, C.H.; Riddle, J.M.; Sabbah, H.N.; Magilligan, D.J., Jr.; Hawkins, E.T. Scanning electron microscopy of operatively excised severely regurgitant floppy mitral valves. *Am. J. Cardiol* **1989**, *64*, 392–394.
36. Mirzaie, M.; Meyer, T.; Schwarz, P.; Lotfi, S.; Rastan, A.; Schondube, F. Ultrastructural alterations in acquired aortic and mitral valve disease as revealed by scanning and transmission electron microscopical investigations. *Ann. Thorac Cardiovasc Surg*. **2002**, *8*, 24–30.
37. Baum, J.; Duffy, H.S. Fibroblasts and Myofibroblasts: What Are We Talking About? *J. Cardiovasc. Pharmacol.* **2011**, *57*, 376–379.
38. Lu, C.C.; Liu, M.M.; Clinton, M.; Culshaw, G.; Argyle, D.J.; Corcoran, B.M. Developmental pathways and endothelial to mesenchymal transition in canine myxomatous mitral valve disease. *Vet. J.* **2015**.
39. Dal-Bianco, J.P.; Aikawa, E.; Bischoff, J.; Guerrero, J.L.; Handschumacher, M.D.; Sullivan, S.; Johnson, B.; Titus, J.S.; Iwamoto, Y.; Wylie-Sears, J.; *et al.* Active Adaptation of the Tethered Mitral Valve Insights Into a Compensatory Mechanism for Functional Mitral Regurgitation. *Circulation* **2009**, *120*, 334.
40. Hagler, M.A.; Hadley, T.M.; Zhang, H.; Mehra, K.; Roos, C.M.; Schaff, H.V.; Suri, R.M.; Miller, J.D. TGF-beta signalling and reactive oxygen species drive fibrosis and matrix remodelling in myxomatous mitral valves. *Cardiovasc Res.* **2013**, *99*, 175–184.
41. Mow, T.; Pedersen, H.D. Increased endothelin-receptor density in myxomatous canine mitral valve leaflets. *J. Cardiovasc. Pharmacol*. **1999**, *34*, 254–260.
42. Olsen, L.H.; Mortensen, K.; Martinussen, T.; Larsson, L.I.; Baandrup, U.; Pedersen, H.D. Increased NADPH-diaphorase activity in canine myxomatous mitral valve leaflets. *J. Comp. Pathol*. **2003**, *129*, 120–130.
43. Kogure, K.Pathology of chronic mitral valvular disease in the dog. *Nihon Juigaku Zasshi*. **1980**, *42*, 323–35.
44. Lu, C.C.; Liu, M.M.; Culshaw, G.; French, A.; Corcoran, B. Comparison of cellular changes in Cavalier King Charles spaniel and mixed breed dogs with myxomatous mitral valve disease. *J. Vet. Cardiol*. **2016**.
45. Hulin, A.; Deroanne, C.F.; Lambert, C.A.; Dumont, B.; Castronovo, V.; Defraigne, J.O.; Nusgens, B.V.; Radermecker, M.A.; Colige, A.C. Metallothionein-dependent up-regulation of TGF-2 participates in the remodelling of the myxomatous mitral valve. *Cardiovascular Res.* **2012**, *93*, 480–489.
46. Aupperle, H.; Marz, I.; Thielebein, J.; Schoon, H.A. Expression of transforming growth factor-beta1, -beta2 and -beta3 in normal and diseased canine mitral valves. *J. Comp. Pathol*. **2008**, *139*, 97–107.
47. Aupperle, H.; Thielebein, J.; Kiefer, B.; Marz, I.; Dinges, G.; Schoon, H.A.; Schubert, A. Expression of Genes Encoding Matrix Metalloproteinases (MMPs) and their Tissue Inhibitors (TIMPs) in Normal and Diseased Canine Mitral Valves. *J. Compar. Pathology* **2009**, *140*, 271–277.
48. Herpin, A.; Lelong, C.; Favrel, P. Transforming growth factor-beta-related proteins: an ancestral and widespread superfamily of cytokines in metazoans. *Dev. Comp. Immunol*. **2004**, *28*, 461–485.
49. Armstrong, E.J.; Bischoff, J. Heart valve development: endothelial cell signaling and differentiation. *Circ. Res.* **2004**, *95*, 459–470.

50. Combs, M.D.; Yutzey, K.E. Heart valve development: regulatory networks in development and disease. *Circ. Res.* **2009**, *105*, 408–421.
51. Horbelt, D.; Denkis, A.; Knaus, P. A portrait of Transforming Growth Factor beta superfamily signalling: Background matters. *Int J. Biochem Cell. Biol*. **2012**, *44*, 469–474.
52. Dobaczewski, M.; Chen, W.; Frangogiannis, N.G. Transforming growth factor (TGF)-beta signaling in cardiac remodeling. *J. Mol. Cell. Cardiol*. **2011**, *51*, 600–606.
53. Pohlers, D.; Brenmoehl, J.; Loffler, I.; Muller, C.K.; Leipner, C.; Schultze-Mosgau, S.; Stallmach, A.; Kinne, R.W.; Wolf, G. TGF-beta and fibrosis in different organs - molecular pathway imprints. *Biochim Biophys Acta* **2009**, *1792*, 746–756.
54. Moesgaard, S.G.; Aupperle, H.; Rajamaki, M.M.; Falk, T.; Rasmussen, C.E.; Zois, N.E.; Olsen, L.H. Matrix metalloproteinases (MMPs), tissue inhibitors of metalloproteinases (TIMPs) and transforming growth factor-beta (TGF-beta) in advanced canine myxomatous mitral valve disease. *Res. Vet. Sci*. **2014**, *97*, 560–567.
55. Obayashi, K.; Miyagawa-Tomita, S.; Matsumoto, H.; Koyama, H.; Nakanishi, T.; Hirose, H. Effects of transforming growth factor-beta 3 and matrix metalloproteinase-3 on the pathogenesis of chronic mitral valvular disease in dogs. *Amer. J. Vet. Res.* **2011**, *72*, 194–202.
56. Waxman, A.S.; Kornreich, B.G.; Gould, R.A.; Moise, N.S.; Butcher, J.T. Interactions between TGFbeta1 and cyclic strain in modulation of myofibroblastic differentiation of canine mitral valve interstitial cells in 3D culture. *J. Vet. Cardiol*. **2012**, *14*, 211–221.
57. Cushing, M.C.; Liao, J.T.; Anseth, K.S. Activation of valvular interstitial cells is mediated by transforming growth factor-beta 1 interactions with matrix molecules. *Matrix Biology* **2005**, *24*, 428–437.
58. Meng, X.M.; Nikolic-Paterson, D.J.; Lan, H.Y. TGF-beta: the master regulator of fibrosis. *Nat. Rev. Nephrol*. **2016**, *12*, 325–338.
59. Disatian, S.; Orton, E.C. Autocrine serotonin and transforming growth factor beta 1 signaling mediates spontaneous myxomatous mitral valve disease. *J. Heart Valve Dis*. **2009**, *18*, 44–51.
60. Connolly, J.M.; Bakay, M.A.; Fulmer, J.T.; Gorman, R.C.; Gorman, J.H., 3rd; Oyama, M.A.; Levy, R.J. Fenfluramine disrupts the mitral valve interstitial cell response to serotonin. *Am. J. Pathol*. **2009**, *175*, 988–997.
61. Oyama, M.A.; Levy, R.J. Insights into Serotonin Signaling Mechanisms Associated with Canine Degenerative Mitral Valve Disease. *J. Vet. Inter.Medic.* **2010**, *24*, 27–36.
62. Porter, K.E.; Turner, N.A. Cardiac fibroblasts: At the heart of myocardial remodeling. *Pharmacology & Therapeutics* **2009**, *123*, 255–278.
63. Atkins, C.E.; Keene, B.W.; Brown, W.A.; Coats, J.R.; Crawford, M.A.; DeFrancesco, T.C.; Edwards, N.J.; Fox, P.R.; Lehmkuhl, L.B.; Luethy, M.W.; *et al.* Results of the veterinary enalapril trial to prove reduction in onset of heart failure in dogs chronically treated with enalapril alone for compensated, naturally occurring mitral valve insufficiency. *J. Am. Vet. Med. Assoc.* **2007**, *231*, 1061–1069.
64. Geirsson, A.; M. Singh, R. Ali, H. Abbas, W. Li, J.A. Sanchez, S. Hashim, and G. Tellides, Modulation of transforming growth factor-beta signaling and extracellular matrix production in myxomatous mitral valves by angiotensin II receptor blockers. *Circulation* **2012**, *126*, S189–S197.
65. Jaffre, F.; Bonnin, P.; Callebert, J.; Debbabi, H.; Setola, V.; Doly, S.; Monassier, L.; Mettauer, B.; Blaxall, B.C.; Launay, J.M.; *et al.* Serotonin and angiotensin receptors in cardiac fibroblasts coregulate adrenergic-dependent cardiac hypertrophy. *Circ. Res.* **2009**, *104*, 113–123.
66. Perez, J.; Diaz, N.; Tandon, I.; Plate, R.; Martindale, C.; Balachandran, K. Elevated Serotonin Interacts with Angiotensin-II to Result in Altered Valve Interstitial Cell Contractility and Remodeling. *Cardiovasc Eng. Technol*. **2017**.
67. Aupperle, H.; Marz, I.; Thielebein, J.; Dinges, G.; Schoon, H.A. Histomorphological findings and expression of matrix metalloproteinases and their tissue specific inhibitors (TIMPs) in normal tricuspid valves and in chronic tricuspid valvular disease in dogs. *Vet. J.* **2010**, *183*, 176–183.
68. Klein, T.; Bischoff, R. Physiology and pathophysiology of matrix metalloproteases. *Amino Acids*, **2011**, *41*, 271–290.
69. Togashi, M.; Tamura, K; Nitta, T.; Ishizaki, M.; Y. Sugisaki; Fukuda, Y. Role of matrix metalloproteinases and their tissue inhibitor of metalloproteinases in myxomatous change of cardiac floppy valves. *Pathol Int*. **2007**, *57*, 251–259.

70. Aupperle, H.; Thielebein, J.; Kiefer, B.; Marz, I.; Dinges, G.; Schoon, H.A. An immunohistochemical study of the role of matrix metalloproteinases and their tissue inhibitors in chronic mitral valvular disease (valvular endocardiosis) in dogs. *Vet. J.* **2009**, *180*, 88–94.
71. Kelwick, R.; Desanlis, I.; Wheeler, G.N.; Edwards, D.R. The ADAMTS (A Disintegrin and Metalloproteinase with Thrombospondin motifs) family. *Genome Biol*. **2015**, *16*, 113.
72. Dupuis, L.E.; McCulloch, D.R.; McGarity, J.D.; Bahan, A.; Wessels, A.; Weber, D.; Diminich, A.M.; Nelson, C.M.; Apte, S.S.; Kern, C.B. Altered versican cleavage in ADAMTS5 deficient mice; a novel etiology of myxomatous valve disease. *Dev. Biol*. **2011**, *357*, 152–164.
73. Kern, C.B.; Wessels, A.; McGarity, J.; Dixon, L.J.; Alston, E.; Argraves, W.S.; Geeting, D.; Nelson, C.M.; Menick, D.R.; Apte, S.S. Reduced versican cleavage due to Adamts9 haploinsufficiency is associated with cardiac and aortic anomalies. *Matrix Biology* **2010**, *29*, 304–316.
74. Grande-Allen, K.J.; Calabro, A.; Gupta, V.; Wight, T.N.; Hascall, V.C.; Vesely, I. Glycosaminoglycans and proteoglycans in normal mitral valve leaflets and chordae: Association with regions of tensile and compressive loading. *Glycobiology* **2004**, *14*, 621–633.
75. Gabriel, L.A.; Wang, L.W.; Bader, H.; Ho, J.C.; Majors, A.K.; Hollyfield, J.G.; Traboulsi, E.I.; Apte, S.S. ADAMTSL4, a secreted glycoprotein widely distributed in the eye, binds fibrillin-1 microfibrils and accelerates microfibril biogenesis. *Invest. Ophthalmol Vis. Sci*, **2012**, *53*, 461–469.
76. Hadian, M.; Corcoran, B.M.; Han, R.I.; Grossmann, J.G.; Bradshaw, J.P. Collagen organization in canine myxomatous mitral valve disease: an x-ray diffraction study. *Biophys. J.* **2007**, *93*, 2472–2476.
77. Tan, H.T.; Lim, T.K.; Richards, A.M.; Kofidis, T.; Teoh, K.L.; Ling, L.H.; Chung, M.C. Unravelling the proteome of degenerative human mitral valves. *Proteomics*, **2015**, *15*, 2934–2944.
78. Radermecker, M.A.; Limet, R.; Lapiere, C.M.; Nusgens, B. Increased mRNA expression of decorin in the prolapsing posterior leaflet of the mitral valve. *Interact. Cardiovasc. Thorac. Surg*. **2003**, *2*, 389–394.
79. Chen, Y.T.; Wang, J.; Wee, A.S.; Yong, Q.W.; Tay, E.L.; Woo, C.C.; Sorokin, V.; Richards, A.M.; Ling, L.H. Differential MicroRNA Expression Profile in Myxomatous Mitral Valve Prolapse and Fibroelastic Deficiency Valves. *Int. J. Mol. Sci*. **2016**, *17*.

© 2017 by the authors. Submitted for possible open access publication under the terms and conditions of the Creative Commons Attribution (CC BY) license (http://creativecommons.org/licenses/by/4.0/).
